# Outcomes after Chemotherapy with WHO Category II Regimen in a Population with High Prevalence of Drug Resistant Tuberculosis

**DOI:** 10.1371/journal.pone.0007954

**Published:** 2009-11-23

**Authors:** Francine Matthys, Leen Rigouts, Vinciane Sizaire, Natalia Vezhnina, Maryvonne Lecoq, Vera Golubeva, Françoise Portaels, Patrick Van der Stuyft, Michael Kimerling

**Affiliations:** 1 Epidemiological and Disease Control Unit, Department of Public Health, Institute of Tropical Medicine, Antwerp, Belgium; 2 Mycobacteriology Unit, Institute of Tropical Medicine, Antwerp, Belgium; 3 Médecins sans Frontières, Brussels, Belgium; 4 HIV/TB Penal System Projects in Central Asian Republics, AIDS Foundation East-West, Alma Ata, Kazakhstan; 5 Laboratory of Mycobacteriology, Colony 33, Mariinsk, Kemorovo, Russia; 6 University of Alabama at Birmingham, Birmingham, Alabama, United States of America; McGill University, Canada

## Abstract

Standard short course chemotherapy is recommended by the World Health Organization to control tuberculosis worldwide. However, in settings with high drug resistance, first line standard regimens are linked with high treatment failure. We evaluated treatment outcomes after standardized chemotherapy with the WHO recommended category II retreatment regimen in a prison with a high prevalence of drug resistant tuberculosis (TB). A cohort of 233 culture positive TB patients was followed through smear microscopy, culture, drug susceptibility testing and DNA fingerprinting at baseline, after 3 months and at the end of treatment. Overall 172 patients (74%) became culture negative, while 43 (18%) remained positive at the end of treatment. Among those 43 cases, 58% of failures were determined to be due to treatment with an inadequate drug regimen and 42% to either an initial mixed infection or re-infection while under treatment. Overall, drug resistance amplification during treatment occurred in 3.4% of the patient cohort. This study demonstrates that treatment failure is linked to initial drug resistance, that amplification of drug resistance occurs, and that mixed infection and re-infection during standard treatment contribute to treatment failure in confined settings with high prevalence of drug resistance.

## Introduction

The notification of tuberculosis (TB) cases in the Russian Federation increased by 7.5% per year during the period 1991 to 1999, reaching 85.2 cases per 100,000 population in 1999 [1]. The deterioration of the general social-economic situation in Russia, the shrinking of the health budget, and the decline of health services during the nineties likely contributed to the resurgence in TB cases [2].

The World Health Organization adopted the DOTS strategy (Directly Observed Therapy, Short Course) as the standard approach to address the global TB epidemic in 1993 [3]. However, TB control during the nineties in Russia did not follow this policy; treatment was not standardized, mass population screening and diagnosis of TB was performed predominately through chest radiography and was often not complemented by bacteriological confirmation. Supply of routine diagnostics and anti-TB drugs became irregular, leading to stock-outs and the erratic treatment of patients. The resulting inadequate treatment of many TB cases likely contributed to the creation of drug resistant TB. The global WHO drug surveillance program has revealed a high prevalence of multi drug resistant TB (MDR TB), defined as resistance to at least isoniazid and rifampicin, in the former Soviet Union [4]. The fourth anti tuberculosis drug resistance survey reports MDR TB estimates for the Russian Federation of 13% among new cases, 48.6% among previously treated cases and 19.4% among combined cases [5] While MDR TB is recognized to be a cause of treatment failure, few published data link drug resistance directly to treatment outcomes. In this article, we present the results of a prospective cohort of newly admitted TB patients in which culture and drug susceptibility testing (DST) was used to document response to the WHO standard Category II (re-treatment) regimen, 2HREZS/1HREZ/5HRE, during 8 months and to assess the limits of such therapy in a setting with a high prevalence of MDR TB. Molecular genotyping was employed to address the issue of mixed infection with a susceptible and resistant strain of M. *tuberculosis* or re-infection by a new strain as a possible mechanism for treatment failure. Finally, we established the amplification of resistance in intermediately resistant strains in those receiving standard TB chemotherapy.

This study was conducted as part of a long-term plan with preparations to initiate MDR-TB therapy with second line drugs in the Kemerovo prison system through the recognized Green Light Committee (GLC) mechanism coordinated by WHO [6].

## Methods

This study was embedded in the ongoing TB treatment programme. All clinical procedures, i.e., sputum sample collection and clinical treatment were performed as part of the routine procedures in place at that time. Sputum smear analysis, culture and DST were routinely done in the local laboratory; subcultures were sent, with the permission of the local health authorities, to the Mycobacteriology laboratory in Antwerp for DST quality control and additional Restriction Fragment Length Polymorphism (RFLP) testing. All patients enrolled in the TB treatment programme gave verbal consent. Demographic, clinical and diagnostic information was retrieved prospectively from the patient clinical file. All data were entered into an unlinked database without individual patient identifiers and analyzed anonymously using Epi-info version 6.2. The study protocol was approved by the local health authorities. At the time the study was carried out, the MSF ethics review board did not yet exist. However, review and approval for publication of the data was obtained retrospectively from the MSF Ethics review board, in June 2008.

The study took place in the penitentiary hospital in Mariinsk in Central Siberia. The prison population in the Kemerovo region was estimated at that time to be nearly 25,000 prisoners distributed over 22 colonies/prisons. Suspected TB cases detected in the general prisons were referred to either the Mariinsk or Novokousnesk TB penitentiary hospital for diagnosis and treatment. Both penitentiary hospitals, conceived for 750 patients, were overcrowded and housed some 1,500 TB patients each. The conditions were very harsh, especially during the long winter months, when outside temperatures may go below minus 40°C and when ventilation inside was extremely poor. The majority of prisoners were packed in ‘wards’ of 20 to 30 persons, slept in bunk beds and had to take turns for a mattress. Because of internal prison security procedures, patients could not be separated according to their infection status or drug resistance pattern and infection control measures were not in place.

Upon arrival to the referral penitentiary hospital, suspected cases were screened through sputum smear analysis, culture and fluoroscopy. Fluoroscopy was used in this prison because films for chest radiography were generally lacking. As many patients had taken undocumented TB drugs before arrival to Colony 33, none of them had however received standard four drug Category I treatment before, and because of the high prevalence of drug resistance and the lack of second line drugs [7], all patients were treated immediately with the WHO recommended Category II (re-treatment) regimen, 2HREZS/1HREZ/5HRE, during 8 months under daily strict direct supervision (H, Isoniazid; R, Rifampicin; E, Ethambutol; Z, Pyrazinamide; S, Streptomycin). All drugs were procured outside Russia and had certificates that guaranteed their quality. Patients also received high energy milk and biscuits as food supplements.

A cohort of 233 consecutive newly admitted patients diagnosed with TB through sputum smear and culture that started treatment between December 1997 and October 1998 were followed until the end of treatment. In order to free space for the suspected TB patients waiting to be transferred from the peripheral prisons, all patients who had completed treatment and had a negative culture were immediately transferred back to other prisons. Those prisons were not accessible for the study team and follow up of these transferred patients was impossible. Relapse cases could therefore not be captured in this study.

Sputum collection practices and DOTS management were those used routinely in the hospital. All patients provided sputum samples for smear microscopy, culture, DST and RFLP analysis at baseline, after three months, and at the completion of treatment. Sputum collection was strictly supervised and included observed mouth rinsing overseen by experienced health staff. Initial cultures were done in the renovated laboratory of Marrinsk and infection control procedures to limit the possibility of laboratory cross-contamination were installed. Subcultures were sent to the Mycobacteriological Unit of the Institute of Tropical Medicine in Antwerp, Belgium, for DST quality control and Deoxyribonucleic acid (DNA)-fingerprinting.

Sputum specimens were decontaminated using the modified Petroff procedure and cultures performed on Löwenstein-Jensen (LJ) medium. [8]. DST was performed on LJ medium by the agar proportion method according to Canatti et al. [9]. All strains were tested for susceptibility to R (40 µg/ml), H (0.2 µg/ml), E (2 µg/ml) and S (4 µg/ml). PZA susceptibility testing was not done. RFLP analysis using the IS6110 probe was performed following standard methods for fingerprinting of *M. tuberculosis* [10]. Genomic DNA was digested with PvuII, electrophoretically fractionated, transferred to Hybond-N^+^ membranes (Amersham) and hybridized with a chemiluminscent-labeled IS6110 3'probe. RFLP profiles were analyzed by means of a computer software program (Gelcomar version 4.1; Applied Maths, Kortrijk, Belgium) using the Dice co-efficient for similarity calculations and the unweighted pair group method with arithmetic averages (UPGMA) for clustering [10]. Two isolates were considered different if they showed 3 or more bands of difference.

## Results

Prior to treatment initiation, isolates from 81 of the 233 patients (34.8%) were susceptible to all four drugs tested (H, R, E, S), 47 (20.2%) were mono drug-resistant, 61 (26.2%) were poly drug-resistant and 44 (18.9%) were MDR-TB. Overall, any resistance to H was 56.6%, to S 51.5%, to E 29.1%, while any resistance to R was 19.3%. Twenty seven patients (11.6%) were mono resistant to H and one patient was mono resistant to R. (Table 1). Patients who had received no or less than one month of therapy before the initiation of category II regimen were more likely to be pan-susceptible than patients who received prior therapy for one month or more (RR = 1.50; 95% C.I. 1.06–2.12) (Table 1). Also, the latter group had significantly more MDR TB than the former (RR = 4.16; 95% C. I. 1.70–10.13).

**Table 1:** Classification of the patients according to drug resistance profiles against the four first-line tuberculosis drugs (H,E,R and S) at time of enrolment (T0).

Classification	Resistant to	<1 month prior therapy (%)	≥1 month therapy (%)	All (%)
Pan-susceptible		36 (44.4)	45 (29.6)	81 (34.8)
Mono-resistant	H	6 (7.4)	21 (13.8)	27 (11.6)
	E	1 (1.2)	2 (1.3)	3 (1.3)
	R	1 (1.2)	0 (0)	1 (0.4)
	S	7 (8.6)	9 (5.9)	16 (6.9)
Subtotal		15 (18.5)	32 (21.1)	47 (20.2)
Poly-resistant*	HE	0 (0)	1 (0.6)	1 (0.4)
	HS	15 (18.5)	16 (10.5)	31 (13.3)
	HES	10 (12.3)	19 (12.5)	29 (12.4)
Subtotal		25 (30.9)	36 (23.7)	61 (26.2)
Multi-resistant°	HRS	0 (0	9 (5.9)	9 (3.9)
	HERS	5 (6.2)	30 (19.7)	35 (15.0)
Subtotal		5 (6.2)	39 (25.7)	44 (18.9)
Total		81 (100)	152 (100)	233 (100)

N = 233 H, Isoniazid; E, Ethambutol; R, Rifampicin; S, Streptomycin.

*Poly-resistant TB defined as resistance to more than one drug but not H and R together.

°Multi-resistant TB defined as resistance to at least H and R.

All 233 TB patients were male prisoners, with a median age of 29 years (range 16–66). Seventy three percent of the patients reported having taken TB drugs before starting Category II treatment. Seventy five percent of patients had a cavity on fluoroscopy and the medium body mass index (BMI) was 19.6 (range 13.4–24.8). (Table 2). The HIV status of patients at the time of TB diagnosis was not independently determined, but the health authorities reported that none of the patients was HIV positive at entry into the prison system. Prior to treatment initiation, demographic and clinical characteristics of the 44 MDR-TB patients before starting treatment were not significantly different compared to the 189 non-MDR TB patients in terms of age, time spent in prison, presence of a cavity on fluoroscopy or BMI. However, MDR-TB patients had been significantly more frequently sentenced than other TB patients, 2.3 versus 1.8 condemnations respectively (p = 0.03).

**Table 2:** Socio-demographic and clinical characteristics of patients at enrolment.

	MDR n = 44	Non MDR n = 189	Total n = 233	P value
Age in years: median (range)	30 (16–66)	29 (19–58)	29 (16–66)	0.59 §
No. of condemnations: median (range)	2.3 (1–12)	1.8 (1–6)	1.9 (1–12)	0.03 §
Months spent in prison: median (range)	34 (7–60)	28 (3–72)	(3–72)	0.06 §
Body mass index*	19.0 (13.4–23.2)	19.7 (15.3–24.8)	(13.4–24.8)	0.06 §
Delay in days°: median (range)	17 (2–58)	18 (4–395)	18 (2–395)	0.62 §
Cavity on fluoroscopy at entry				
Yes no. (%)	35 (79.5)	139 (73.5)	174 (74.7)	
No no. (%)	9 (2.0)	49 (25.9)	58 (24.9)	0.66 #
Unknown no. (%)	0	1 (0.5)	1 (0.4)	

* Body mass index is calculated as the weight in kilograms divided by the square of the height in meters.

° Delay: time in days between admission to Colony 33 and the start of treatment.

§ t-test; # Chi square test.

Of the 233 TB patients who started treatment, 172 (73.8%) became culture negative at the end of treatment, 43 (18.4%) remained culture positive, 1 patient died and 17 patients (7.3%) had treatment interruption because they were released from prison while still on therapy (Table 3). Excluding patients who interrupted treatment, 92% of the pan-susceptible TB patients, 91% of the mono drug-resistant, 86% of the poly drug-resistant and 37% of the MDR-TB cases had a negative culture at the end of the treatment.

**Table 3:** Treatment outcome of the patients at the end of Category II treatment according to their DST profile at start of treatment.

	Total N (%)	Culture – N (%)	Culture + N (%)	Died N (%)	Interrupted N (%)
DST profile					
Pan-susceptible	81 (34.8)	71 (87.7)	6 (7.4)	0	4 (4.9)
Mono-resistant	47 (20.2)	38 (80.9)	4 (8.5)	0	5 (10.6)
Poly-resistant	61 (26.2)	48 (78.7)	8 (13.1)	0	5 (8.2)
MDR	44 (18.9)	15 (34.1)	25 (56.8)	1 (2.3)	3 (6.8)
Total	233 (100)	172 (73.8)	43 (18.4)	1 (0.5)	17 (7.3)

Pan-susceptible: susceptible to all four drugs H,E,R,S.

Mono-resistant: resistance to one drug.

Poly-resistant: resistant to at least two drugs, simultaneous resistance to H and R excluded.

MDR: Multi-drug resistant: resistance to at least H and R.

Table 4 compares the DST results of the 43 patients who remained culture positive at the end of the treatment (T8) with the DST results at baseline (before the start of treatment, T0) and after the initial 3 months of treatment (T3). Patients are classified according to the drug resistance profile before starting treatment (T0). Table 4 also shows the comparison of the RFLP patterns of the isolates obtained at baseline (T0) and after treatment completion (T8) for each patient. Twenty five patients had strains with identical RFLP patterns before, during and after treatment and can be considered as not responding to the chemotherapy regimen they received. Seventeen out of the 25 patients were already resistant to all 4 drugs before treatment, while the eight other patients were poly drug-resistant before starting treatment and acquired resistance to the drugs they were still susceptible to at the start of treatment. Seven of them acquired drug resistance during the first three months; one patient had a negative culture at T3. Amplification occurred in 6 out of the 56 of initial poly drug-resistant (10.7%) but not MDR-TB patients, excluding the defaulters.

**Table 4:** Drug susceptibility testing results before the start (T0), after 3 months (T3), and at the end of treatment (T8) for patients remaining culture positive at the end of a full treatment course with comparison of RFLP results at T0 and T8.

No. of Patients	Resistance pattern at T0	N	Resistance pattern at T3	N	Resistance pattern at T8	Identical RFLP	Changed RFLP	Interpretation
6	Susceptible	1	Culture –	1	susceptible		1	re-infection or laboratory error
		1	Culture –	1	E		1	re-infection or laboratory error
		3	Culture –	3	HERS		3	re-infection or mixed infection
		1	HES	1	HERS		1	re-infection+ acquired R resist or mixed infection
1	S	1	Culture –	1	HERS		1	re-infection or mixed infection
3	H	2	Culture –	2	HERS		2	re-infection or mixed infection
		1	HERS	1	HERS		1	re-infection or mixed infection
4	HS	1	Culture –	1	Susceptible		1	re-infection or laboratory error
		1	Culture –	1	HRS		1	re-infection or mixed infection
		1	Culture –	1	HERS		1	re-infection or mixed infection
		1	HERS	1	HERS	1		acquired ER resistance
4	HES	3	HERS	3	HERS	3		acquired R resistance
		1	Culture –	1	HERS	1		acquired R resistance
4	HRS	3	HERS	3	HERS	3		acquired E resistance
		1	HRS	1	HERS		1	re-infection or mixed infection*
21	HERS	9	Culture –	9	HERS	7		treatment failures
							1	mixed or re-infection
							1	mixed infection§
		9	HERS	9	HERS	7		treatment failures
							1	mixed infection or re-infection°
							1	mixed or re-infection**
		3	n/a	3	HERS	3		treatment failures
43	Total	43		43		25	18	43

Number of patients  = 43.

RFLP, Restriction Fragment Length Polymorphism; H, Isoniazid; R, Rifampicin; E, Ethambutol; S, Streptomycin; n/a  =  not available.

* T0 and T3 isolates identical; T3 and T8 isolates different.

§ Both RFLP profiles were unique.

° Intermediate isolate (T3) showed different RFLP.

** RFLP at T8 not available, but RFLP patterns at T0 and T3 were different.

Seventeen patients had a strain with different RFLP pattern before and after treatment. Two cases carried a fully susceptible strain before treatment, became smear and culture negative after three months of treatment, but had a different strain, one pan-susceptible and one mono-resistant to ethambutol, at the end of the treatment. A third patient with an initial strain resistant to H and S became culture negative after 3 months of treatment but had a pan-susceptible strain cultured at the end of treatment. The 14 remaining patients with a different RFLP isolate profile at T0, (four pan-susceptible, four mono-resistant, two HS resistant and four MDR strains) and T8 had a MDR strain at the end of treatment. For one patient the RFLP pattern at month 8 was not available; however, the RFLP patterns at T0 and T3 were different.

## Discussion

The result of this study showing a MDR-TB prevalence of 19% among this inmate cohort in Mariinsk is very high and consistent with previously published Colony 33 data from 1997 where the prevalence of MDR TB was 22% [7]. Similarly high levels of MDR-TB were reported among prisoners in Azerbaijan and Georgia during the same time period [12], [13]. While a history of inadequate TB treatment may be the original underlying cause of drug resistance, once MDR-TB is created, it becomes a source of primary infection and possibly re-infection while under treatment, especially in an overcrowded, poorly ventilated prison environment. Transmission of drug-resistant strains has been documented in other congregate settings such as hospitals and prisons and in HIV [14], [15], [16]. Mono resistance to H was more frequent (11.6%) than mono resistance against R (0.4%). However, since PZA susceptibility testing was not performed, this gives not the full picture.

The finding that MDR-TB was positively associated with the number of condemnations, and thus passage through the pre-trial centres where prisoners were kept for long periods of time in extremely overcrowded and harsh living conditions while awaiting their trial, suggests that MDR-TB strains may have been transmitted in these pre-trial centres. High rates of TB have been documented in a pre-trial detention centre in Kemerovo [17]. The HIV status of the patients was not confirmed independently, but the fact that no other AIDS related opportunistic infections were detected and the low case fatality offer assurance that HIV infection was not a problem.

Seven percent of the 233 patients interrupted therapy because they were released from prison before their conviction release date. Although they were referred to public health services to complete treatment, it is unknown if they continued their treatment. All other patients, with the exception of the patient who died, completed their treatment. Adherence was very high, which may be partly explained by the previous experience patients had witnessing daily deaths in TB afflicted inmates and by the fact that the prison inmate leadership strongly supported the Colony 33 DOTS program.

The failure rate among the patients who completed treatment was 20%. High failure rates of 29% after completion of Category I or II have also been reported in a prison population in Azerbaijan, where the MDR TB prevalence was 23% [12]. Treatment success, i.e., a negative culture at the end of treatment, for pan-susceptible, mono-resistant and poly-resistant non-MDR patients who completed treatment can be considered good as they achieved or surpassed the WHO target of 85%. However, the culture conversion rate at the end of treatment of 37% for the MDR TB patients is extremely poor and approaches the spontaneous cure rate of tuberculosis seen in the pre-chemotherapy days. Treatment failure was clearly linked to the initial drug resistance pattern and cannot be ascribed to a lack of treatment supervision, poor quality drugs or other programmatic problems. The overall treatment success of 74% based on a negative culture at treatment completion (T8) should be interpreted cautiously as patient follow up beyond treatment completion was not feasible. This represents a limitation of the study and the observed treatment success is most probably an over-estimate of the eventual outcome since subsequent TB relapse can not be excluded.

Indeed, Migliori and colleagues reported 28% relapses among MDR TB patients who had achieved treatment success on standard short course chemotherapy within a median time to relapse of 8 months after completion of treatment [18], [19].

The resistance amplification rate of 10.7% detected in the initially poly-resistant patients may be an underestimate since relapse TB was not captured due to the impossibility to follow up patients once treatment finished. Several investigators have reported amplification of resistance to additional agents while receiving WHO recommended regimens of category I and II [20]. However few reports on amplified drug resistance have been supported by concurrent RFLP analysis to differentiate resistance amplification from mixed or re-infection. Cox and colleagues reported that 17% of poly drug-resistant, but not multidrug-resistant strains of patients with the same RFLP profile, acquired additional drug resistance during short-course directly observed treatment [21]. Unlike the study reported by Cox, drug resistance testing for pyrazinamide was not done in the current study. Additional drug resistance was acquired early during the first three months of therapy, consistent with the findings from the Cox study [21].

Thirty five percent of the patients with a positive culture at the end of treatment had a changed RFLP profile. Three patients, two with an initially fully susceptible strain and one resistant to H and S, became culture negative after 3 months of treatment but had either a pan-susceptible strain (two patients) or a strain resistant to E (one patient), cultured at the end of treatment. These patients may have been re-infected with a new strain or may represent episodes of laboratory error, mislabelling or cross-contamination. The remaining 15 patients with changed RFLP isolate profile between T0 and T8 were likely re-infected or had mixed infections. Overcrowding and the extreme living conditions in this penitentiary hospital together with the lack of infectious control measures may have favoured re-infection. Such conditions are however rarely encountered in other non-congregate and congregate settings. A retrospective analysis of treatment outcomes in Tomsk [22] found that patients who began treatment in a hospital setting and who were hospitalized during their treatment had a substantially higher risk of developing MDR TB than those who were treated as outpatients. Nosocomial re-infection with a MDR strain may be a plausible explanation.

However, it is impossible to determine with certainty if a patient with a different strain at the end of treatment represents an initial mixed infection, i.e., an infection with two different strains of *M. tuberculosis* or a re-infection with another strain during treatment. This study performed DNA fingerprinting but part of the attributed re-infections may in fact be initial mixed infections. It has been shown, using highly specific polymerase chain reaction (PCR)-based genotyping methods, that in TB patients in a high incidence setting, the same sputum sample contains different M. tuberculosis strains [23]. The presence of different M. tuberculosis strains when analyzing different pre-treatment sputum samples from the same patient was also demonstrated [24]. Mixed infections with a drug susceptible and a drug resistant strain at the beginning of treatment may thus be an alternative explanation for the observed changes in strains and resistance patterns before and at the end of treatment. The pre-dominance of the drug susceptible strains before treatment could be due to a difference of fitness and growth between the drug susceptible and the drug resistant strain [25]. The undetected drug-resistant strain could then have been selected under the pressure of treatment with first line drugs. [26], [27].

Excluding the 3 cases associated with a possible laboratory error, all patients who remained culture positive at the end of the treatment, except one who was still susceptible to E, showed resistance to all four first-line TB drugs tested. This finding has important implications for any proposed re-treatment regimen in settings with high prevalence of drug resistance. Adding a single drug to a failing regimen or a regimen likely to fail must absolutely be avoided exception. The high rate of drug resistance found at baseline before the start of treatment (T0) also raises the question of what drugs an initial treatment regimen should include in such settings. These issues have previously been raised elsewhere [11], [12]. Thus, in settings with a high prevalence of drug resistance, it is mandatory to perform routine DST, not only for all re-treatment cases but also for newly diagnosed cases, in order to guide and tailor treatment accordingly and to avoid resistance amplification and further transmission of resistant strains. For timely results, rapid culture and PCR based drug resistance testing methods are needed as drug resistance is acquired early during treatment.

In conclusion, this study re-confirms the importance of resistance amplification and the need for DST to guide treatment in settings with high prevalence of drug resistance. The use of an inadequate chemotherapy regimen was the cause of treatment failure (same RFLP profile) in 58% of the patients who remained culture positive at the end of treatment whereas 42% of the failures are due to an initial mixed infection or a re-infection (changed RFLP profile) while being on treatment. A mixed or re-infection is the reason for treatment failure in all initial pan-susceptible and mono-resistant TB patients that remained culture positive at the end of treatment.

Although re-infection has been documented in the case of recurrent TB in several studies, very few have documented the possibility of re-infection while on TB therapy. This finding is mainly important for settings with a high prevalence of MDR TB and underscores the of the need for adopting comprehensive measures to prevent re-infection. Specific and strict infection control measures must be implemented in congregate settings and especially in prisons with high prevalence of (MDR) TB. In the wake of the emergence of XDR TB, the current HIV epidemic and the recommendations of the WHO-convened XDR TB Task Force in 2006, such measures are of even greater concern. [28].
